# Supplementary material for: Efficacy of home-based and inpatient treatment for children and adolescents in psychiatric crisis: A systematic review protocol
Source: PLoS One. 2025 Mar 10;20(3):e0318792. doi: 10.1371/journal.pone.0318792 (PMC11893119; doi:10.1371/journal.pone.0318792)
Supplement: S2 Table — (PDF) [file pone.0318792.s002.pdf]

Supporting Information

Search Strategy

**Table 1 Search strategy Medline via PubMed**
1. child* OR pediatric* OR kid OR kids OR (german [Language]) AND (kind*) OR infant*
2. adolesc* OR juvenil* OR youth* OR teenage* OR “young adult*”
3. child [MeSH]
4. adolescent [MeSH]
5. infant [MeSH]
6. adolescent behavior [MeSH]
7. child behavior [MeSH]
8. young adult [MeSH]
9. adolescent psychiatry [MeSH]
10. OR/1-9
11. "psychiatric cris*" OR "mental health cris*" OR "psychiatrische krise*" OR "psychische krise*"
12. "psychiatric emergenc*" OR "mental health emergenc*"
13. "severe mental illness*" OR "severe mental disorder*" OR "severe psychiatric illness*" OR "severe psychiatric disorder*"
14. "acute mental illness*" OR "acute mental disorder*" OR "acute psychiatric disorder*"OR “acute psychiatric illness*”
15. “mental health problems” OR “psychiatric disorder*”
16. "suicidal ideation*" OR "attempted suicide*" OR suicid* OR "self-harm*" OR suizid* OR selbstverletz*
17. suicide, attempted [MeSH]

18. suicidal ideation [MeSH]
19. self-injurious behavior [MeSH]
20. OR/11-19
21. hometreat* OR “home treat*” OR “home therapy” OR “home care”
22. “inpatient equivalent” OR “ward equivalent” OR stationsaquivalent* OR StaB OR StaeB OR StäB OR “aufsuchende behandlung*”
23. mst OR “multisystemic treatment*” OR “multisystemic therap*” OR multisystemisch* OR multisystemic OR multi- systemic
24. “intensive community treatment*” OR “intensive support service*” OR “intensive case management”
25. “crisis resolution team*” OR “crisis team*” OR CRT OR “psychiatrische krisenintervention*”
26. “family centered treatment*” OR FCT
27. “assertive community treat*” OR “assertive outreach”
28. “mobile treatment”
29. “alternative to hospitalization” OR “alternative to hospitalisation”
30. “discharge support” OR “discharge service*”
31. home care service [MeSH]
32. home care services, hospital based [MeSH]
33. community mental health centers [MeSH]
34. community mental health services [MeSH]
35. mobile health units [Mesh]
36. community mental health services [Mesh]
37. crisis intervention [Mesh]
38. emergency services, psychiatric [Mesh])

39. OR/21-38
40. "inpatient treatment*" OR "psychiatric inpatient care" OR "inpatient mental health care"
41. "psychiatric hospitalization" OR "psychiatric hospitalisation" OR "mental health hospitalization" OR "mental health hospitalisation"
42. "psychiatric admission*"
43. "psychiatric ward" OR "psychiatric unit*" OR "psychiatric facilit*" OR "psychiatric hospital*" OR "psychiatric clinic*" OR "mental health hospital*" OR "mental health clinic*" OR "mental health facilit*" OR "mental health unit*"
44. stationär* OR stationar* OR stationaer*
45. inpatients [MeSH]
46. child psychiatry [MeSH]
47. adolescent psychiatry [MeSH]
48. emergency services, psychiatric [Mesh]
49. hospitals, psychiatric [Mesh]
50. mental health services [Mesh]
51. OR/40-50
52. 10 AND 20 AND 39 AND 51
